# Autonomic Nervous System Function Following Prenatal Opiate Exposure

**DOI:** 10.3389/fped.2013.00027

**Published:** 2013-10-17

**Authors:** Matthew Todd Hambleton, Eric W. Reynolds, Thitinart Sithisarn, Stuart J. Traxel, Abhijit R. Patwardhan, Timothy N. Crawford, Marta S. Mendiondo, Henrietta S. Bada

**Affiliations:** ^1^Department of Pediatrics, University of Kentucky, Lexington, KY, USA; ^2^Department of Biomedical Engineering, University of Kentucky, Lexington, KY, USA; ^3^Department of Biostatistics, College of Public Health, University of Kentucky, Lexington, KY, USA

**Keywords:** heart rate variability, autonomic nervous system, prenatal opiate, power spectral analysis, neonatal abstinence syndrome

## Abstract

*In utero* exposure to opiates may affect autonomic functioning of the fetus and newborn. We investigated heart rate variability (HRV) as a measure of autonomic stability in prenatal opiate-exposed neonates (*n* = 14) and in control term infants (*n* = 10). Electrocardiographic data during both non-nutritive and nutritive sucking were evaluated for RR intervals, heart rate (HR), standard deviation of the consecutive RR intervals (SDRR), standard deviation of the differences of consecutive RR intervals (SDDRR), and the power spectral densities in low and high frequency bands. In controls, mean HR increased significantly, 143–161 per min (*p* = 0.002), with a trend toward a decrease in RR intervals from non-nutritive to nutritive sucking; these measures did not change significantly among exposed infants. Compared to controls, exposed infants demonstrated significantly greater HRV or greater mean SDRR and SDDRR during non-nutritive period (*p* < 0.01), greater mean SDDRR during nutritive sucking (*p* = 0.02), and higher powers in the low and high frequency bands during nutritive feedings. Our findings suggest that prenatal opiate exposure may be associated with changes in autonomic nervous system (ANS) functioning involving both sympathetic and parasympathetic branches. Future studies are needed to examine the effects of prenatal opiate exposure on ANS function.

## Introduction

Heart rate variability (HRV) has been used as a measure of autonomic nervous system (ANS) function for over 30 years. Studies, such as by Siassi et al. (1) in 1979, examined HRV in both time and frequency domains to evaluate the health of the ANS. The ANS controls involuntary physiologic responses through its two main branches, the sympathetic and parasympathetic systems. These branches often function in opposition to each other to achieve homeostasis of involuntary functions such as breathing, digestion, and excretion. In cardiac muscle, the sympathetic nervous system functions to increase both rate and force of contraction, while the parasympathetic nervous system functions primarily to decrease heart rate (HR). In vascular smooth muscle, the sympathetic system primarily causes vascular wall contraction, while the parasympathetic system causes vascular relaxation. The derived HRV measures from beat by beat fluctuation in cardiovascular variables, are used as an index of ANS function (2–8) reflecting the status of sympathetic and parasympathetic balance. HRV is usually assessed in two ways: by time domain analysis and by frequency domain analysis. The calculations in the time domain analysis are based on the statistical derivations from consecutive RR intervals (interbeat intervals) measured from the electrocardiogram (EKG) tracings. The RR intervals are sometimes referred to as the heart period. In the frequency domain, the periodic fluctuations in HR are examined as these fluctuations are affected by temperature, the baro-receptor reflex, and respirations. The HR fluctuation that is equal to the respiratory rate from the inspiratory inhibition of the vagus nerve is noted in the high frequency (HF) region and has been referred to as the respiratory sinus arrhythmia (RSA) or vagal tone. In most studies, the low frequency (LF) band usually includes the regions from 0 to 0.2 Hz and the HF band between 0.2 and 1.5 Hz (9, 10).

Heart rate variability has served as an indicator of ANS system functioning in clinical settings, in subjects of all ages. In the fetus, HRV has been used as one of the indicators of fetal well being (11). In the newborn infants, the HRV has been studied in active and quiet sleep (12), in intrauterine growth restriction (13), in assessment of pain (14), and in sepsis (15). In older infants, autonomic changes such as brief increases in HR and RSA were noted to parallel increases in negative emotions during tasks eliciting frustration (16). The evaluation of the ANS system in the newborn is of interest since studies have suggested that physiological measures may predict neurobehavior, temperament, and later childhood outcomes (17, 18). In older children, ANS changes as shown by changes in heart period and vagal tone or RSA were associated with behavior problems (19, 20).

The ANS functioning has been evaluated in infants with prenatal substance exposure such as cocaine and tobacco. Compared to infants with no prenatal tobacco exposure, exposed infants had higher HR and RSA (21). Neonates with prenatal cocaine exposure showed autonomic alterations; they had higher HRV; i.e., higher total power in frequency domain and higher vagal tone (22). However, other investigators found similar RR interval dynamics between those with prenatal cocaine exposure and controls during quiet and active sleep (23). In one report, infants with prenatal cocaine exposure had delayed but prolonged response in HR or decrease in HRV during orthostatic stress (6).

There are few studies on ANS functioning following prenatal opiate exposure. Experimental studies indicate that prenatal opiate exposure may affect both the developing fetal hypothalamic-pituitary-adrenal axis and the sympathetic adrenal medullary axis (24, 25), and thereby resulting in alteration in not only cortisol response but also norepinephrine and adrenalin response affecting HR and HRV. These effects on the hypothalamic-pituitary-adrenal axis and the sympathetic adrenal medullary axis were evident even at later ages. Also in adult rats with perinatal exposure to the opiate, oxycodone, changes in blood pressure, and HR responses to acute stress were different from animals with no prenatal opiate exposure (26).

Clinical studies also suggest that prenatal opiate exposure may result in alterations in ANS functioning. Fetuses of mothers on methadone treatment had decreased baseline HR, HRV, and acceleration (27). Following prenatal opiate exposure, infants had increases in HR as well as associated decreases in vagal tone (5); these changes in cardiac measures occurred with drug withdrawal manifestations or neonatal abstinence syndrome (NAS). Other investigators also found that abnormal HR patterns, e.g., increase in base line HR and beat-to-beat variability, were associated with tremors and irritability in infants with NAS (28). Older children with prenatal opiate exposure appeared to have impairment in ANS functioning as indicated by increased vagal tone reactivity in the presence of task with increased attentional demand (29).

With the increasing prevalence of opiate use in women of child bearing age (30), the number of reported NAS cases also is on the rise (31). Infants with NAS manifest central nervous system and ANS signs; they may have increase in HR, increase in respiratory rate, and have difficulty with feeding (suck-swallow-breath interaction) (32). Investigators reported on abnormalities in the feeding patterns, changes in length and frequency of sucking bursts, less rhythmic swallowing, and abnormalities in respiratory control (33, 34); these may affect both sympathetic and parasympathetic functioning. Yet there are few reports on the ANS functioning after prenatal opiate exposure. A more detailed investigation of ANS functioning in exposed children will help better understand the underlying physiological changes associated with the clinical syndrome and its treatment. Furthermore, characterization of neonatal ANS functioning in prenatal opiate exposure may have the potential for determining a relationship between early ANS functioning and infant behavior regulation and or later outcomes (17, 18).

To assess the ANS status in neonates, investigators have elicited changes in ANS functioning using different stimuli (e.g., nutritive sucking, non-nutritive suck with use of a pacifier, or by tilt or orthostatic changes) (4, 6, 35). Since feeding is a routine activity for newborn infants, we used nutritive sucking to elicit possible changes in ANS functioning and determined measures of HRV in the time domain and the rhythms about the HRV in the frequency domain. We hypothesized that prenatal opiate exposure would be associated with increased activity in the sympathetic and parasympathetic systems.

## Materials and Methods

This study is a part of a prospective larger study on feeding using a sample of convenience, with Institutional Review Board approval. The complete protocol has been published elsewhere (36). Subject enrollment was done after birth. Informed consent was obtained from a parent. For this ancillary study, we evaluated HRV from recordings of two groups of term infants: a group with prenatal opiate exposure with NAS symptoms and a control group with no prenatal opiate exposure. Although term gestation was a prerequisite for enrollment, no matching was done as to birth weight or gender. The opiate exposure was identified by maternal history of opiate use and or by a positive opiate screen from infant’s urine or meconium. All infants admitted had urine and meconium drug screening. As part of our clinical practice, infants with prenatal opiate exposure were assessed every 3–4 h for symptoms of withdrawal using the Finnegan scoring system (37). In this scoring system, 21 items are scored according to severity. Items scored include central nervous system and autonomic nervous manifestations, including signs of respiratory and gastrointestinal dysfunction. Examples of items scored are tremors, muscle tone, sweating, fever, yawning, mottling, nasal stuffiness, sneezing, nasal flaring, tachypnea, excessive feeding, poor feeding, regurgitation or vomiting, loose or watery stools, skin excoriations, and etc. For this study we noted the Finnegan NAS scores (37) of each exposed infant documented by the bedside nurse within a few hours before the study, as well as the highest score at anytime prior to the study.

### Recording procedures

Infants were monitored with a 3-lead EKG. Two electrodes were placed on the upper chest on each side, below the clavicle and in proximity to the left and right arm. The third electrode was placed on left lower extremity, proximal to the ankle. All data sets were collected at times the infant was scheduled to feed per nursing protocols. EKG signals (Model #90623A, Space Labs Inc., Redmond, WA, USA) were acquired from the RS-232 port and analog signals converted to digital data. Signals were digitized using a commercial data acquisition system at a rate of 600 samples per second (Model #DI-706, DATAQ Instruments, Akron, OH, USA). The study procedure coincided with scheduled feedings, with infants in awake state. Prior to the data acquisition, each infant was swaddled and held by the research nurse to prepare for feeding. EKG recording started when a pacifier was given for at least 1 min to stimulate infant sucking and readiness for bottle feeding. After non-nutritive sucking, the infant was given his/her feeding (nutritive sucking). EKG recording continued until the infant consumed the prescribed volume or for no longer than 15 min.

### Analysis of EKG signals

A custom-made program in MATLAB (MathWorks, Natick, MA, USA) was developed to analyze EKG data. With this software, consecutive peaks of R waves were identified from the EKG tracings. After identification of R peaks, the RR intervals (millisecond) were computed. Data segments with artifacts with three or less unidentifiable R peaks were removed and values linearly interpolated using the preceding RR and the succeeding RR after the deleted artifact segment. When data segments had more than three unidentifiable R peaks, the segments were deleted and removed from the analysis. Of the data segments, 4% contained artifacts and were not analyzed. One of the authors (Stuart J. Traxel) ran the custom software for R peak detection and EKG data edits; he was masked to the study group assignment.

Electrocardiogram signals were examined in the time and frequency domains. In the time domain, variation in HR was examined from the intervals of consecutive QRS, measured between peaks or RR. The beat-to-beat changes are the results of the complex interaction of vagal, sympathetic, and other influences on the heart. Therefore in this study, the HRV measures included the RR intervals, HR (beats per minute) derived from the RR, and the statistical derivations namely: standard deviation of the consecutive RRs intervals (SDRR), and standard deviation of the difference between consecutive RR intervals (SDDRR). For each infant during each minute of non-nutritive and nutritive sucking, the means for the RR intervals, HR, SDRR, and SDDRR were calculated to compare these time domain variables between controls and opiate-exposed infants. Since during nutritive sucking, the EKG recordings for each infant lasted for 15 min, we also compared the time domain variables between controls and opiate-exposed infants during a 5-min period.

In the frequency domain, periodic changes in heart pattern within each given frequency band over a range of frequencies were determined from spectral power display. To extract information at particular frequencies, RR intervals were equi-sampled and then filtered using a digital low-pass filter with a cut-off of 12.5 Hz. After filtering, data were sub-sampled at 25 Hz since components at frequencies much larger than 1.5 Hz are infrequently used in power spectral analysis (PSA) of HRV. Sub-sampled data were segmented into 120 s sections and each section was zero-meaned and multiplied by a Hanning window. The Fast Fourier Transform (FFT) was computed for all data segments. Spectral density was then computed by averaging the FFT of all data segments for each infant. During non-nutritive sucking, the infant’s EKG data that were <120 s in duration were zero-padded to equal 120 s in duration to increase frequency resolution obtained at low frequencies. EKG data that were longer than 120 s were divided into segments using a 50% overlap.

For the frequency domain analysis, we performed PSA of the RR intervals, representing power at each frequency for each category of infants (controls versus opiate-exposed by non-nutritive and nutritive sucking), allowing for a visual interpretation of which frequencies in each domain (high and low) are more greatly affected. LF power was calculated for each infant as the area underneath the spectral density curve between 0.01 and 0.2 Hz. HF power was calculated similarly for frequencies between 0.2 and 1.5 Hz. These calculations summarize the impact on changes to RR variability at low and high frequencies generally, measured for each category of infants in power per Hertz. Except for a lower cut-off at the low end of the LF band, we used frequency bands similar to those used by other investigators (9, 10, 38–40). The amplitude of power in the LF band is influenced by both sympathetic and parasympathetic systems. The two components of the power spectrum in the LF are related to thermoregulatory fluctuations in vasomotor tone and the frequency response of the baro-receptor reflex (12, 41). The LF may also have the component of fluctuations due to breath amplitude modulation (38). The spectral power in the HF band has its peak centered at the respiratory frequency, reflecting the sinus arrhythmia caused by respirations. Thus, the peak at HF may shift with changes in respiratory rate. Fluctuations in the HF are modulated by the parasympathetic branch of the ANS (9, 41, 42).

### Statistical methods

Descriptive statistics included frequencies, medians, means, standard error of the mean (SE), and proportions. Normality was assessed for each of the measures. As to the time domain measures, we compared the 1-min means of RR, HR, SDRR, and SDDRR during non-nutritive sucking and nutritive sucking within and between controls and the exposed group, using mixed linear models. The 5-min time domain variables between groups during nutritive sucking were compared using the *T* test. In exposed infants, the use of Pearson’s Correlation assessed the linear association between NAS scores and each of the time domain measures during non-nutritive and nutritive sucking. We also performed mixed linear models to compare the frequency domain measures between the controls and exposed groups during non-nutritive and nutritive periods. The method adjusts for multiple comparisons using Tukey–Kramer method. For all analysis, a *p*-value <0.05 was considered significant. For all analyses we used SAS version 9.3 (Cary, NC, USA).

## Results

### Enrolled subjects

Enrollment consisted of 24 subjects, 10 controls, and 14 opiate-exposed infants. Mothers of control infants did not use opiates and 2 of 10 used tobacco during pregnancy. Seven of 14 mothers in the exposed group were on methadone and the remaining used oxycodone or hydrocodone or both. Three opiate using mothers also smoked during pregnancy. Mean (SE) birth weight of controls, 3221 (176) g, did not differ from that of the opiate-exposed group, 3045 (125) g. Similarly, mean gestational ages were comparable, 38.7 (0.28) and 39.4 (0.38) weeks for controls and exposed groups respectively. Median 1- and 5-min Apgar scores for term controls were 9 and 10, respectively. Median 1- and 5-min Apgar scores were both 9 for exposed infants. Of the controls, 40% had transient respiratory distress on admission compared to 21% in the exposed group. The respiratory distress was attributed to delayed adaptation and resolved within a few hours of admission. No infant had respiratory distress at the time of the study procedure. There were more males in the control group, 80 versus 36% in the exposed group (*p* < 0.05). Infants were studied within 1 week of birth except for four opiate-exposed, who were studied during the second week of life. In opiate-exposed infants, the Finnegan scores (37) obtained a few hours before the physiological data acquisition ranged from 4 to 20 (median, 11). However, the highest scores recorded for the study subjects at any time prior to the study procedure ranged from 12 to 22 (median, 16), scores high enough for infants to need pharmacologic treatment.

**Figure 1:**
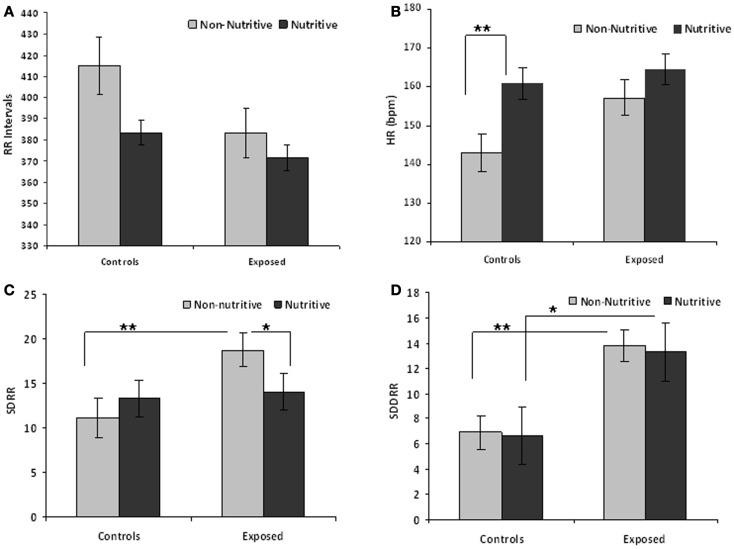
**(A–D)** Show the means (SE) of time domain variables for controls and exposed subjects during non-nutritive (pacifier) and nutritive sucking. Also significant differences are indicated as follows: *p < 0.05; **p < 0.01. **(A)** Compares the mean RR (millisecond) between controls and exposed infants during non-nutritive and nutritive periods. **(B–D)** Respectively show the HR (beats per minute), SDRR, and SDDRR changes between controls and opiate-exposed infants during non-nutritive and nutritive sucking.

Table 1 compares the means (SE) of the measures in time domain. The *t* values and adjusted *p*-values from multiple comparisons are also shown. Figures 1A–D illustrate the results from the time domain analysis. During non-nutritive sucking, mean RR (SE) did not differ between controls and exposed infants, 415.0 (13.2) and 383.1 (10.1) ms, respectively. In controls, RR decreased marginally during nutritive sucking to 383.5 (13.2) ms, *p* = 0.08, while no significant decrease was noted in the exposed infants (Figure 1A). Also, during non-nutritive sucking, a marginal difference was noted in HR between groups; mean (SE) in controls was 143 (5) versus 157 (4) per min in exposed infants (Figure 1B). HR increased significantly during nutritive sucking in controls to 161 (4.7) per min (*p* = 0.002), but not in exposed infants. Figure 1C shows that during non-nutritive sucking, the exposed infants had higher SDRR values compared to controls, mean (SE) of 20.6 (2.5) versus 11.0 (1.3); estimated mean (SE) difference was −7.5(2.7), *p* = 0.006. Exposed infants also showed a significant decrease in SDRR, from mean (SE) of 20.6 (2.5) during non-nutritive to 14.1 (2.5) during nutritive sucking, but no significant change occurred in controls. As to SDDRR, no significant change occurred in controls or in exposed infants from non-nutritive to nutritive periods (Figure 1D). However, those exposed had significantly higher SDDRR values during non-nutritive (*p* < 0.002) and nutritive (*p* < 0.02) sucking compared to controls.

**Table 1:** **Comparison of time domain variables (RR interval, HR, SDRR, and SDDRR) and frequency domain variables (LF and HF) during non-nutritive and nutritive sucking between controls and exposed infants^a^**.

	RR interval	HR	SDRR	SDDRR	LF	HF
	
	Mean (SE)					
Controls
Non-nutritive	415.0 (13.2)	143.0 (4.8)	11.03 (1.3)	6.93 (1.3)	0.2201 (0.08)	0.2093 (0.12)
Nutritive	383.5 (13.2)	161.0 (4.7)	10.8 (1.3)	6.67 (1.3)	0.3119 (0.08)	0.3204 (0.12)
*t* Value	1.75	−3.20	0.12	0.17	−0.87	−0.78
*p*-Value	0.0838	0.002	0.9051	0.8626	0.3938	0.4453
Exposed
Non-nutritive	383.1 (10.1)	157.1 (4.0)	20.6 (2.5)	13.87 (2.3)	0.4135 (0.07)	0.4155 (0.10)
Nutritive	371.6 (10.1)	164.5 (4.0)	14.1 (2.5)	13.31 (2.3)	0.6066 (0.07)	0.8230 (0.10)
*t* Value	0.83	−1.33	2.03	0.18	−2.16	−3.38
*p*-Value	0.410	0.1876	0.0448	0.8595	0.0415	0.0027
Controls versus exposed
Non-nutritive
*t* Value	1.63	−1.76	−2.82	−3.38	−1.88	−1.29
*p*-Value	0.1061	0.0812	0.0059	0.0011	0.0738	0.2120
Nutritive
*t* Value	1.19	−1.35	−0.23	−2.59	−2.86	−3.13
*p*-Value	0.2385	0.1803	0.8170	0.0111	0.0091	0.0048

^a^Means (SE), *t* value, and adjusted *p*-value are shown for multiple comparisons (Tukey–Kramer).

We also found differences in the 5-min time domain measures during nutritive sucking between controls and exposed infants. Those exposed had a significantly shorter RR, mean (SE) of 359.9 (7.9) compared to controls, who had mean (SE) of 387.5 (10.3), *p* = 0.04. HR during the nutritive period for exposed infants was 167.7 (3.5) per min, higher than 155.8 (4.1) per min in controls. The mean (SE) of the SDRR and SDDRR measures did not differ between controls and exposed infants during the nutritive period.

Among exposed infants, there was no correlation between the Finnegan scores recorded within 4 h of the data acquisition and any of the time domain measures. However, the highest scores recorded prior to the study procedure correlated significantly, but only during the nutritive sucking periods, with the following time domain measures: RR (*r* = 0.24, *p* < 0.02), HR (*r* = −0.22, *p* < 0.03), and SDDRR (*r* = 0.22, *p* < 0.03). Therefore, during nutritive feeding, longer RR intervals, lower HR, and higher SDDRR correlated with increasing severity of NAS.

### Frequency domain analysis

**Figure 2:**
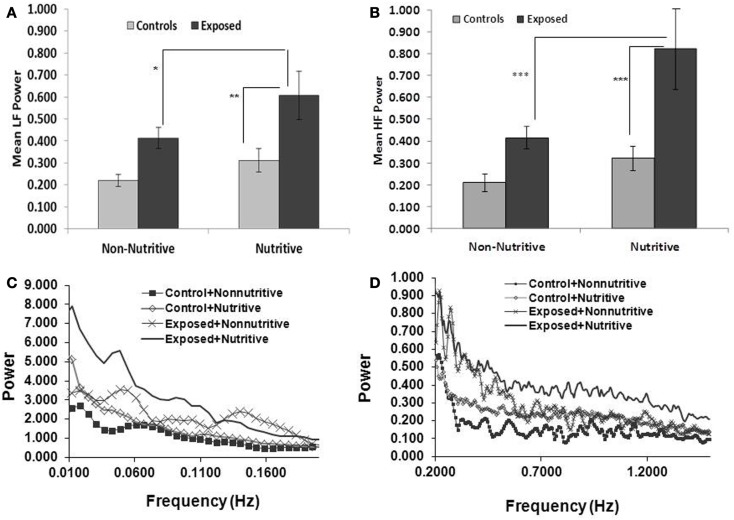
**(A,B)** (Top panels) show the mean (SE) power per Hertz for the LF and HF bands respectively, comparing the controls and opiate-exposed infants during non-nutritive and nutritive sucking. Significant differences are indicated as follows: *p < 0.05; **p < 0.01; ***p < 0.005. In both controls and exposed, LF and HF power per Hertz increases from non-nutritive to nutritive periods but significant change occurred only in exposed in the LF domain (*p* < 0.05) and HF domain (*p* < 0.005). Compared to controls, exposed infants have higher LF (*p* < 0.05) and HF (*p* < 0.03) power per Hertz during nutritive sucking. **(C,D)** (Bottom panels) illustrate the plots of power spectral densities against the LF band of 0.01–0.2 Hz and HF band of 0.2–1.5 Hz. The controls during non-nutritive sucking have the lowest power and the exposed have the highest power in both LF and HF bands. Note that the scale for power against frequency in **(D)** is expanded for HF to provide more details.

Shown in Figures 2A,B and in Table 1 are the means (SE) of power for the LF (0.01–0.2 Hz) and HF (0.2–2.5 Hz), in the control and exposed infants during non-nutritive and nutritive periods. The power in LF and HF increased from non-nutritive to nutritive sucking in both groups, but significant increase was noted only in the exposed with a mean difference of −0.19, *p* = 0.04 for the LF and mean difference of −0.4075, *p* = 0.0027 for HF. Both LF and HF powers were significantly greater in the exposed group during nutritive sucking compared to controls. Shown in Figures 2C,D, are the power spectral densities in the LF and HF domains plotted against frequencies of 0.01–0.2 and 0.2–1.5 Hz, respectively. The displays of PSA in both LF and HF bands show that controls during non-nutritive sucking had the lowest power while the exposed during nutritive sucking had the highest power in both the LF and HF bands. The LF to HF ratios did not differ between non-nutritive sucking and nutritive sucking within groups or between groups.

## Discussion

To our knowledge, this is the first report on examining ANS functioning after prenatal opiate exposure during non-nutritive and nutritive sucking. Our results suggest that prenatal opiate exposure may affect neonatal ANS functioning in both sympathetic and parasympathetic branches. Compared to controls, exposed infants showed a greater HRV by some of the time domain measures. In exposed infants during nutritive sucking, HRV was higher with higher Finnegan scores. Further, PSA indicated a prominent increase in activity for both sympathetic and parasympathetic branches in exposed infants compared to controls during nutritive sucking.

The findings of increase in HR and decrease in RR from non-nutritive to nutritive sucking may be related to increased autonomic output as evidenced by the gustatory hypothesis. The gustatory hypothesis proposes that due to shared nervous system nuclei, digestive processes could have concurrent cardiac changes (43). Lipsitt and co-investigators (44) related their findings in newborns of the associated increase in HR during sucking with liquid (sucrose) compared to sucking with no liquid (non-nutritive) to adaptive gustatory phenomenon; i.e., a slower sucking rate when fed a “savored substance.” Previous studies evaluated HR and HRV in normal, healthy infants during non-nutritive and nutritive sucking with variable results (3, 4, 7, 44, 45). Our results are in line with findings by Cohen et al. (3, 4), as well as Portales et al. (35), who noted an increase in autonomic function during nutritive feeding from baseline in healthy infants. We noted similar changes in our controls, i.e., increase in HR with nutritive sucking, but with minimal and insignificant change in those with opiate exposure. Although we did not have measurements prior to non-nutritive sucking, other investigators (4, 7) found no significant change between baseline HR and the HR determined while sucking a pacifier. The marginally elevated baseline HR of the opiate-exposed may be explained by an increase in sympathetic activity, decrease in parasympathetic activity, or a combination of the two. Changes during embryologic development to cholinergic receptor sites or other nerve synapse sites with opioid receptors may affect the ANS functioning (46, 47). Also, ANS alterations in exposed newborns may be related to the effect of opiate on the ontogeny of the stress axis with prenatal exposure effects on the hypothalamic-pituitary-adrenal axis interacting with ANS functioning (25).

Jansson et al. (5) examined heart periods in neonates born with perinatal methadone exposure. They found longer average heart periods on days 1 and 2, than the mean RR intervals in our study. Differences in the methodology of their study from ours include an earlier monitoring period (day of life 1–3), no control group, EKG recording at sleep state, and less severe Finnegan scores or better pharmacological control than our exposed infants.

The significant differences in the time domain measures, SDRR and SDDRR, during non-nutritive and nutritive sucking between controls and exposed infants deserve explanation. The SDDRR, reflecting the beat-to-beat HRV is influenced by changes in respiratory rate or pattern, changes in vasomotor tone, changes in inotropic function, as well as changes in the suck-swallow-breath mechanisms affecting vagal tone. LaGasse and others (33) showed that prenatal drug exposure leads to abnormalities in the feeding patterns and behaviors of neonates, such as changes in length and frequency of sucking bursts. Gewolb et al. (34) found faster swallow rate, less rhythmic swallowing, and mild abnormalities in respiratory control in opiate-exposed neonates. Therefore, it can be surmised that our findings of autonomic changes after prenatal opiate exposure, with a stimulus such as feeding, may in part be related to a disorganized oral motor skills. The disorganized oral motor skills and abnormalities in suck-swallow-breath rhythms may affect not only RSA but also the associated small tidal volumes changes occurring with breathing. These tidal volume variations affect specifically powers in the LF bands (48), while changes in vagal tone affect the HF powers.

The LF region in our study includes frequencies correlating with rhythms attributable to changes in vasomotor tone and the functioning of the sympathetic system (9). The HF band frequencies correlate to events, such as changes in respiration and HR, reflecting the activity of the parasympathetic system (9, 39, 49). Our findings of significant differences in power in both LF and HF bands between the controls and opiate-exposed neonates suggest an association between exposure and increased activity of both sympathetic and parasympathetic systems.

We adjusted the cutoffs of the LF and HF bands by considering the higher HR and breathing rates in infants relative to those in adults. Spectral divisions for neonates between 0.04 and 0.15 Hz for LF and 0.4–1.5 for HF have been reported (50). Our bands for LF and HF respectively, were from 0.01 to 0.2 and from 0.2 to 1.5 Hz. The HF band in our analysis is similar to those used by others which included a low cut-off of 0.2 Hz (10, 38–40). We did not exclude the 0.15–0.4 Hz region and we used a slightly lower end for LF. The reason for using the slightly lower end of LF was a concern about resolution, given that we were working with smaller data segments. Given that the primary contribution to the HF band is the RSA, which results from gating of vagal activity by discharges in the phrenic nerve, we did not want to exclude contributions to the HF from breaths that may have fallen within the 0.2–0.4 Hz region. Drug-exposed infants are reported to have disorganized suck and swallow rhythms that may affect regularity and rate of breathing (33, 34).

There are limitations to our study. Although we had at least 5 min of EKG recording in our subjects during nutritive sucking, we only had a 1-min of recording during the non-nutritive sucking of the pacifier and therefore shorter than standard recommendation (51). However, with both shorter and longer duration of recording, we noted differences in the time domain measures between controls and exposed.

We did eliminate artifacts and ectopic beats after data acquisition. Nevertheless, the artifact rejection process itself introduces errors in any situation, especially more so when data lengths are limited, specifically in the non-nutritive sucking segments. The difference in age at enrollment between our two groups may introduce bias. But a previous study reported a constant influence of feeding on HR for the first 6 months of life in healthy infants (7). Therefore, the differences in our findings between exposed and controls suggest a link to prenatal opiate exposure rather than post natal age.

The small number of infants in our study limits generalizability of our findings. Nagy et al. found male newborns to have lower baseline HR than female infants (52). A higher proportion of male infants did comprise our subjects, but our small study number precluded a meaningful analysis to determine gender effects on HRV.

Investigators have reported that maternal smoking affects fetal and neonatal HR patterns (21, 53). However, the small number of subjects in our study rendered us unable to examine any interaction between opiate use and other drugs including tobacco use during pregnancy. We also did not quantify use of tobacco or other drugs during pregnancy and thus we were not able to correlate ANS function with amount of drug use.

In summary, our findings suggest that prenatal opiate exposure may have an effect on the functioning of the sympathetic and parasympathetic systems. Therefore, determination of ANS functioning following *in utero* opiate exposure needs to be further explored. With a longer duration of data acquisition (51), a customized approach for spectral frequency band designations (50), and a large sample, the effects of opiate exposure on ANS balance can be better elucidated and the effects of gender, other drug exposures, and confounders can be evaluated. The correlation between HRV and the severity of NAS based on Finnegan scoring in our study gives rationale for future studies to examine ANS functioning for a role as an adjunct in monitoring neurobehavior of infants with NAS.

## Conflict of Interest Statement

The authors declare that the research was conducted in the absence of any commercial or financial relationships that could be construed as a potential conflict of interest.

## Abbreviations

ANS, autonomic nervous system; EKG, electrocardiogram; FFT, fast Fourier transform; HF, high frequency; HR, heart rate; HRV, heart rate variability; LF, low frequency; NAS, neonatal abstinence syndrome; PSA, power spectral analysis; RSA, respiratory sinus arrhythmia; SDDRR, standard deviation of the differences of consecutive RR intervals in a subject; SDRR, standard deviation of the consecutive RR intervals.
